# 5-Hydroxytryptamine Modulates Migration, Cytokine and Chemokine Release and T-Cell Priming Capacity of Dendritic Cells In Vitro and In Vivo

**DOI:** 10.1371/journal.pone.0006453

**Published:** 2009-07-31

**Authors:** Tobias Müller, Thorsten Dürk, Britta Blumenthal, Melanie Grimm, Sanja Cicko, Elisabeth Panther, Stephan Sorichter, Yared Herouy, Francesco Di Virgilio, Davide Ferrari, Johannes Norgauer, Marco Idzko

**Affiliations:** 1 Department of Pneumology, University Hospital Freiburg, Freiburg, Germany; 2 Department of Experimental and Diagnostic Medicine, Section of General Pathology, Interdisciplinary Center for the Study of Inflammation, University of Ferrara, Ferrara, Italy; 3 Department of Dermatology, University Hospital Freiburg, Freiburg, Germany; 4 Department of Dermatology, University Hospital Jena, Jena, Germany; Charité-Universitätsmedizin Berlin, Germany

## Abstract

Beside its well described role in the central and peripheral nervous system 5-hydroxytryptamine (5-HT), commonly known as serotonin, is also a potent immuno-modulator. Serotoninergic receptors (5-HTR) are expressed by a broad range of inflammatory cell types, including dendritic cells (DCs). In this study, we aimed to further characterize the immuno-biological properties of serotoninergic receptors on human monocyte-derived DCs. 5-HT was able to induce oriented migration in immature but not in LPS-matured DCs via activation of 5-HTR_1_ and 5-HTR_2_ receptor subtypes. Accordingly, 5-HT also increased migration of pulmonary DCs to draining lymph nodes in vivo. By binding to 5-HTR_3_, 5-HTR_4_ and 5-HTR_7_ receptors, 5-HT up-regulated production of the pro-inflammatory cytokine IL-6. Additionally, 5-HT influenced chemokine release by human monocyte-derived DCs: production of the potent Th1 chemoattractant IP-10/CXCL10 was inhibited in mature DCs, whereas CCL22/MDC secretion was up-regulated in both immature and mature DCs. Furthermore, DCs matured in the presence of 5-HT switched to a high IL-10 and low IL-12p70 secreting phenotype. Consistently, 5-HT favoured the outcome of a Th2 immune response both in vitro and in vivo. In summary, our study shows that 5-HT is a potent regulator of human dendritic cell function, and that targeting serotoninergic receptors might be a promising approach for the treatment of inflammatory disorders.

## Introduction

Outside the central nervous systems 5-hydroxytryptamine (5-HT), commonly known as serotonin, is found mainly in platelets and can be released during platelet aggregation. Additionally, it has been shown that mast cells are also able to store and release 5-HT, e.g. after cross-linking of membrane bound IgEs by allergens [1]–[3].

The wide variety of biological activities and the complexity of pharmacologic activities mediated by 5-HT, are due to the existence of different classes of serotoninergic receptors (5-HTR) [4]: the 5-HTR_1_ subgroup consists of at least five subtypes namely 5-HTR_1A_, 5-HTR_1B_, 5-HTR_1D_, 5-HTR_1E_, and 5-HTR_1F_. 5-HTR_1A_ interacts with several G proteins, eliciting different responses [5]. 5-HTR_1B_ and 5-HTR_1D_ are coupled to formation of inositol phosphates through interaction with pertussis toxin–sensitive G_i/o_ and pertussis toxin–insensitive G_q_ proteins [6]. The G protein–coupled 5-HTR_2_ includes three different subtypes: 5-HTR_2A_, 5-HTR_2B_, and 5-HTR_2C_ [7]. The 5-HTR_3_ is a ligand-gated cation channel triggering depolarization of the plasma membrane through activation of Na^+^ and K^+^ fluxes [8]. The 5-HTR_4_ receptor has two splice variants (5-HTR_4a_ and 5-HTR_4b_) [9]. The heptahelical 5-HTR_5_ subtype is less characterized [10]. 5-HTR_6_ and 5-HTR_7_ are linked to Gs protein–mediated stimulation of adenylyl cyclase [11], [12].

Apart from its well-characterized function as a neurotransmitter, 5-HT has been reported to be a potent immunoregulator: Stimulation of serotoninergic receptors in human monocytes or lung epithelial cells is associated with secretion of pro-inflammatory cytokines [13], [14]. Additionally, 5-HT plays a prominent role in T-cell activation and in the interaction between T-cells and dendritic cells [15], [16]. Furthermore, 5-HT has chemotactic activity for eosinophils and mast cells [17], [18]. Consistently, elevated serotonin levels have been detected in inflammatory diseases such as bronchial asthma where a strong correlation between disease severity and free plasma serotonin has been found [19]. Methysergid, an antagonist at 5-HTR_2_ receptors, was able to inhibit airway inflammation and remodelling in an animal model of asthma [20].

Dendritic cells (DCs) are the most potent antigen presenting cells, originating from hematopoietic stem cells [21], [22]. Immature DCs are specialized in capturing and taking up antigens. DC maturation can be induced by danger signals such as bacterial endotoxin [23], [24], or pro-inflammatory cytokines, e.g. TNF-α. During maturation MHCII and co-stimulatory molecules on the cell surface are up-regulated and different cytokines and chemokines are produced. Mature DCs migrate to secondary lymphoid organs where they interact with naive T-cells, resulting in either Th1- or Th2-dominated immune responses [21]. DCs also produce several proinflammatory cytokines such as TNF-α, IL-1β, IL-6, and IL-8 profoundly affecting the outcome of inflammatory reactions [16], [25]. Furthermore DCs have been shown to be essential in the pathogenesis of inflammatory processes such as bronchial asthma [26]–[28]. Recently, we were able to demonstrate the functional expression of 5-HTR on human DCs, linked to intracellular Ca^2+^-signalling and cytokine secretion [25]. However, the influence of 5-HT on migration, production of chemokines such as CXCL10/IP-10 or CCL22/MDC, and T-cell polarization capacity has not been investigated yet.

In this study, we demonstrated that 5-HT is a chemoattractant for immature but not LPS-matured DC in vitro and in vivo, via activation of 5-HTR_1B_ and 5HTR_2_ subtypes, while it modulate the secretion of IL-6, CXCL10, CCL22 and the T-cell polarization capacity of mature DC, via activation of 5-HTR_4_ and 5-HTR_7_ subtypes.

## Materials and Methods

### Reagents

5-Hydroxytryptamine (5-HT), N-Methyl-5-Hydroxytryptamine (2Me5HT, 5-HTR_3_ agonist), R-(-)-DOI-hydrochloride (DOI, 5-HTR_2_ agonist), Ketanserin (5-HT_2_ antagonist), and LPS were obtained from Sigma-Aldrich (Deisenhofen, Germany). 5-Carboxamidotryptamine maleate (5-CT, 5-HTR_1_ agonist), BRL-54443 (5-HT_1E/F_ agonist), 8-Hydroxy-DPAT-hydrobromide (8-HDPAT, 5-HTR_7/1A_ agonist), GR 55562 (5-HT_1B_ antagonist), Anpirtoline hydrochloride (AnHCL, 5-HTR_1B_ agonist), RS67333 hydrochloride (5-HT_4_ agonist), RS39604 hydrochloride (5-HT_4_ antagonist) and SB-269970 (5-HT_7_ antagonist) hydrochloride were purchased from Tocris (Bristol, UK).

### Preparation of human DC

Peripheral mononuclear cells were separated from buffy coats using a Ficoll gradient. After separation, the leukocyte-containing pellet was resuspended in 2 ml PBS containing 0.15% EDTA and 0.5% BSA. Cells were separated with anti-CD14 mAb-coated MicroBeads using Macs single use separation columns from Miltenyi Biotec (Bergisch Gladbach, Germany). The CD14^+^ cells were cultured for 5 days in RPMI 1640 medium containing 10% FCS, 1% glutamine, 50 IU/ml penicillin, 50 µg/ml streptomycin, 1,000 U/ml IL-4, and 10,000 U/ml GM-CSF (Promocell, Heidelberg, Germany) at 37°C in an humidified atmosphere with 5% CO_2_. These cells were CD14^neg^, CD1a^pos^, CD80^low^, CD83^low^, CD86^low^, and >95% CD115^high^ and are also referred as immature DC. Maturation of DC was induced by incubation for 24 h in the presence of 3 µg/ml lipopolysaccharide (LPS; Sigma-Aldrich). Mature DC were >95% CD80^high^, CD86^high^ CD83^high^ and CD115^low^. Monoclonal Abs and their respective isotype controls were from Coulter-Immunotech, Krefeld, Germany.

### Migration assay

Experiments were performed in triplicate using 48-well Transwell chambers (Nucleopore, Tübingen, Germany). Buffer or stimuli were added into the lower compartment wells. Thereafter a 10 µm polycarbonate membrane with a pore size of 5 µm (Nuclepore) was placed over the wells. DC (10^5^ cells/well) were added to the upper compartment and incubated at 37°C for 90 min in a humidified atmosphere. After removing the cells from the upper side of the membrane by wiping over a profiled rubber, migrated DC on the lower side of the membrane were fixed in methanol and stained with hematoxylin. Migrated DC were counted in 5 randomly chosen high-power (x400) fields and a mean value for each sample was calculated. The chemotactic index was calculated as the ratio between DC migrated in the presence and in the absence of stimuli.

### Cytokine assays

IL-10 and IL-12p70 were measured in DC supernatants by ELISA using matched pair of mAbs from BD PharMingen (San Diego, CA). Quantikine human TNF-α ELISA was from R&D Systems (Minneapolis, USA). CXCL10 and CCL17 were measured in DC supernatants by ELISA using matched pair of mAbs and ELISA kit from BD PharMingen and R&D Systems, respectively. Release of IFN-γ and IL-5 from T cells was detected using matched pairs of mAbs (BD PharMingen). Samples were assayed in triplicate for each condition.

### T cell differentiation assay

CD4^+^ T lymphocytes were separated (>95% CD4^+^ cells) from the heavy density fraction (50–60%) of Ficoll gradients (Amersham-Pharmacia Biotech) performed on PBMC, followed by immunomagnetic depletion using a mixture of anti-HLA-DR, anti-CD19 and anti-CD8 mAb conjugated beads (Dynal, Oslo, Norway). Allogeneic naive T cells were purified (>95% CD45RA^+^) by incubation of CD4^+^ cells with anti-CD45RO mAb followed by a goat anti-mouse Ig coupled to immunomagnetic beads, and then co-cultured (10^6^ cells/well) with DC (5×10^4^ cells/well) in a 24-well plate in 1640 RPMI medium supplemented with 5% human serum. After 5d IFN-γ, IL-5 and IL-13 release were determined by ELISA.

### Animal studies

#### Mice

BALB/c mice (6–8 weeks old) were purchased from Harlan (Zeist, The Netherlands). OVA-TCR transgenic mice (DO11.10) on a BALB/c background were bred at the animal facilities at the University Hospital Freiburg.

### Effect of 5-HT on the T-cell priming capacity of DCs

Ova-specific naive T cells (1×10^7^) purified from DO11.10 TCR transgenic mice were injected intravenously in the lateral tail vein of BALB/c mice (day 0). On day 2, the mice received an i.t. injection of 1×10^6^ OVA-pulsed DCs, 5-HT/OVA-pulsed DCs, or control unpulsed DCs. On day 6, MLNs were collected and homogenized. For cytokine measurement, MLN cells were plated in round-bottom 96-well plates (1×10^6^ cells/ml in RPMI1640/5% FCS) and re-stimulated with OVA (10 mg/ml) for 4 days. IL-4, IL-5, IL-13, and IFN-γ were assayed in supernatants by ELISA (R&D Systems, Minneapolis, USA; eBioscience Inc., San Diego, CA).

### Effect of 5-HT on DC migration

To address migration of lung DCs 80 µl of FITC-OVA (10 mg/ml), with or without 5-HT (was administered intratracheally under direct vision through the opening vocal cords using a 18-gauge polyurethane catheter connected to the outlet of a micropipette as previously described (4). Control mice received 80 µl of PBS/DMSO. At 24–36 h after injection, migrating DCs were enumerated in the mediastinal LN as CD11c+MHCII+ cells carrying FITC+ material.

In all experiments, dead cells were excluded form analysis using propidium iodide. Analysis was performed on a FacsCalibur flow cytometer, using Cellquest and FlowJo software.

### Ethics statement

Experiments with human cells were reviewed and approved by the ethics committee of the University hospital Freiburg. Animal experiments were approved by the local animal ethics committee (Regierungspräsidium Freiburg) and performed according to the respective guidelines.

### Statistical analysis

For all experiments, the statistical significance of differences between samples was calculated using the ANOVA, Bonferroni comparison test. Differences were considered significant if p<0.05.

## Results

### 5-HT induces migration of immature but not mature DCs via activation of 5-HTR_1_ and 5HTR_2_ receptor subtypes

**Figure 1:**
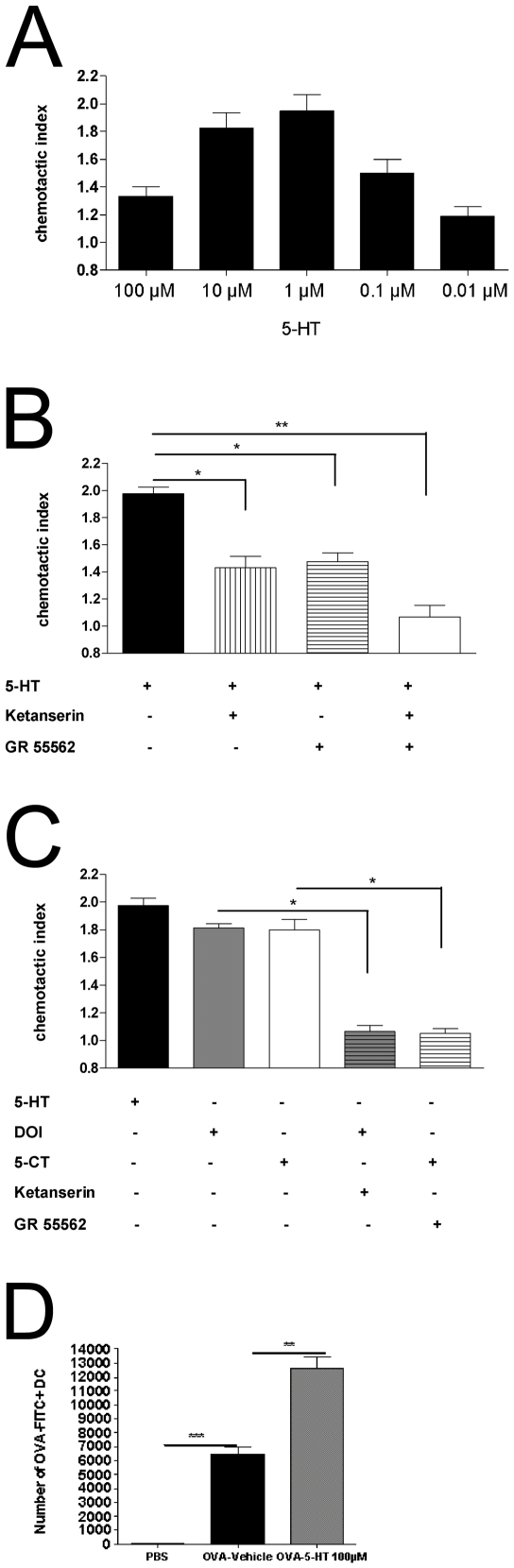
5-HT induces migration of immature DCs. (A) DCs were exposed to the indicated concentrations of 5-HT for 90 min at 37°C in a Boyden chamber as described in material and methods. The chemotactic index was calculated. Data are means±SEM (*n* = 5). (B) Cells were pre-treated with the 5-HTR_1B_ antagonist G55562, 5-HTR_2_ antagonist ketanserin (both 100 nM) or vehicle. Migration in response to 5-HT (1 µM) was determined as described above and the chemotactic index was calculated as described above ([Fig. 1B]). Data are means±SEM (*n* = 5). (C) Cells were pre-treated with the 5-HTR_1B_ antagonist G 55562, the 5-HTR_2_ antagonist ketanserin (both 100 nM) or vehicle. Migration in response to 5-HT, the 5-HTR_2_ agonist DOI (10 µM), and the 5-HTR_1_ agonist 5-CT (10 µM) was determined and the chemotactic index was calculated as described above ([Fig. 1C]). Data are means±SEM (*n* = 5) *p<0.05, **p<0.01, ***p<0.001. (D) Mice were injected intratracheally with OVA-FITC (10 mg/ml) together with 5-HT (100 µM) or vehicle. One day later, the presence of OVA-FITC carrying CD11c^+^MHCII^+^ DCs in mediastinal lymph nodes was analyzed by flow cytometry.

It has recently been reported that 5-HT is a potent chemoattractant for human mast cells and eosinophils, via activation of 5-HTR_1_ or 5HTR_2_ receptor subtypes respectively [17], [18]. To investigate the ability of 5-HT to induce migration of DCs, immature and LPS-matured DCs were stimulated with increasing concentrations of 5-HT. As shown in Figure 1A 5-HT elicited a typical dose-dependent bell-shaped chemotactic response for immature DCs, while it failed to induce migration in LPS-matured DCs.

To determine the 5-HTR-subtypes involved in the 5-HT induced migration, immature DC where stimulated with 5-HT (1 µM) in the presence of the 5-HTR_1B_ antagonist GR55562 (100 nM), 5-HTR_2_ antagonist ketanserin or vehicle. Both antagonists alone were able to partially inhibit 5-HT mediated migration, while when applied in combination they completely abolished this response (Fig. 1B), suggesting the involvement of 5HTR_1B_ and 5-HTR_2A_ subtypes.

Stimulation with the 5-HTR_1_ agonist 5-CT or the 5-HTR_2_ agonist DOI also resulted in migration of immature DCs. This response was blocked by pre-incubation with GR55562 or ketanserin (Fig. 1C).

### 5-HT induces migration of DCs to mediastinal lymph nodes

To test whether 5-HT is also a chemotaxin for DCs in vivo, fluorescently labelled OVA was injected intratracheally together with 5-HT or vehicle. The transport of the large molecule FITC-OVA is an exclusive function of lung-derived DCs, being able to transport this complex across the epithelial tight junction barrier. The number of MHCII^+^CD11c^+^ DCs carrying fluorescent FITC-OVA cargo was enumerated in the MLNs 1 day after instillation, and, as shown in Figure 1D, 5-HT markedly increased the number of DCs in mediastinal lymph nodes.

### 5-HT modulates cytokine and chemokine production in mDCs

We previously reported that 5-HT can enhance the production of the pro-inflammatory cytokines IL-8 and IL-1β via activation of 5-HTR_3_, 5-HTR_4_ and 5-HTR_7_ subtypes in human monocyte-derived dendritic cells [25]. We therefore were interested if 5-HT is also able to influence secretion of IL-6, IP-10, MDC IL-10 or IL-12p70 in immature and mature DCs. Cells were stimulated for 24 h and cytokine content in the supernatants was analyzed by ELISA.

**Figure 2:**
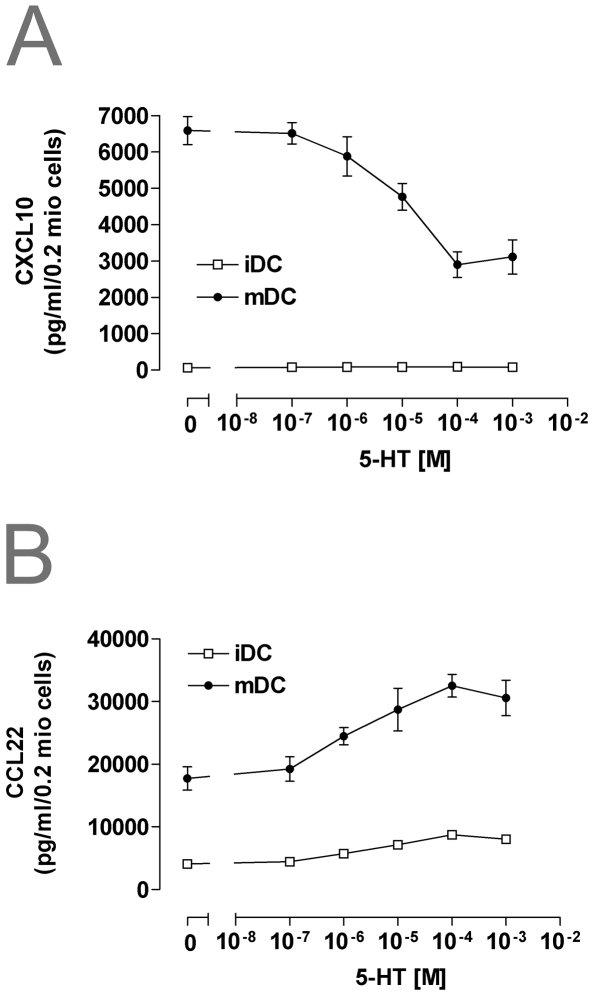
5-HT inhibits the release of IP 10/CXCL10 and stimulates MDC/CCL22 secretion in DCs. Immature and mature DCs were left untreated or were stimulated with the indicated concentration of 5-HT for 24 h (A–B). CXCL10 and CCL22 release was measured by ELISA. The results are expressed as mean pg/ml±SEM (n = 4).

Immature DCs produce high levels of CCL22/MDC and very low levels of IP-10/CXCL10, whereas in LPS-matured DCs the secretion of both CCL22 and CXCL10 is up-regulated (Fig. 2). When DCs were treated with LPS in the presence of 5-HT, a dose dependent inhibition of CXCL10 production was observed (Fig. 2A), while the production of CCL22 was increased. In addition 5-HT also enhanced the secretion of CCL22 in immature DCs (Fig. 2B).

**Figure 3:**
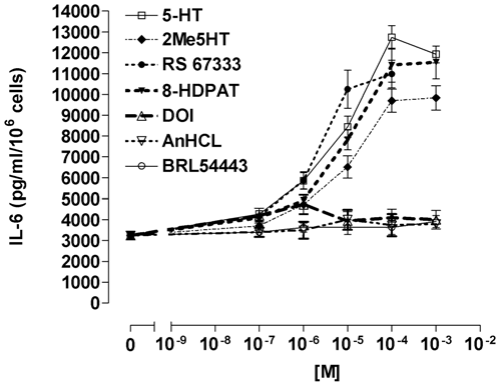
Stimulation of serotoninergic receptors induces secretion of IL-6 in mature DCs. Mature DCs were stimulated with the indicated concentrations of 5-HT or isotype receptor agonists. Supernatants were collected 24 h after stimulation and IL-6 concentration was measured by ELISA. Results are given as mean ± SEM (n  =  4).

The proinflammatory cytokine IL-6 is involved in both acute inflammation and chronic tissue remodelling. As shown in Figure 3, 5-HT, the 5-HTR_3_-agonist 2Me5-HT, the 5-HTR_4_ agonist RS 67333 and the 5-HTR_7/1A_ agonist 8-HDPAT increased IL-6 release of in maturing DCs.

**Figure 4:**
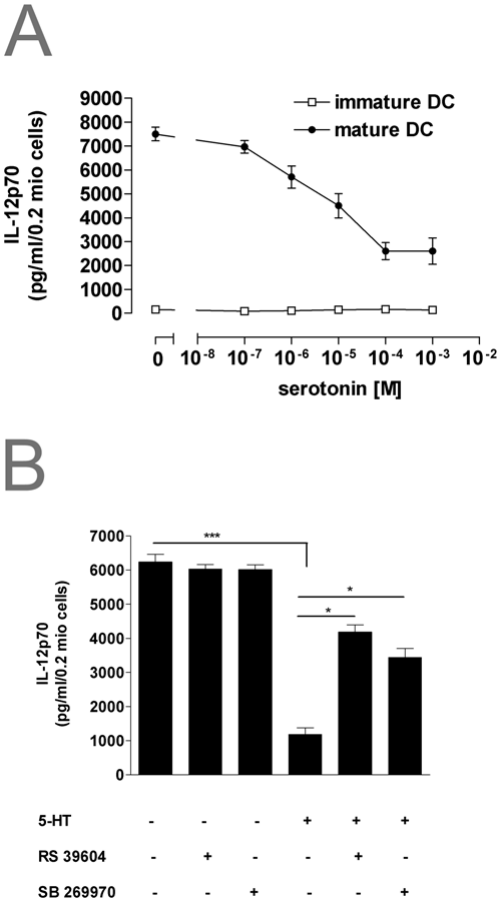
5-HT modulates IL-12p70 production of human monocyte-derived DCs. (A) Immature and mature DCs were stimulated with the indicated concentration of 5-HT concentrations. Supernatants were collected 24 h after stimulation and cytokine content was measured by ELISA. Results are given as mean±SEM (n = 4). (B) Mature DCs were preincubated with 10^−7^ M of the selective 5-HTR_4_ antagonist RS39604 or the 5-HTR_7_ antagonist SB269970 30 min prior to stimulation with 5-HT. 24 hours later production of IL-12p70 was measured by ELISA. Data are means±SE (*n* = 5) *p<0.05, **p<0.01, ***p<0.001.

**Figure 5:**
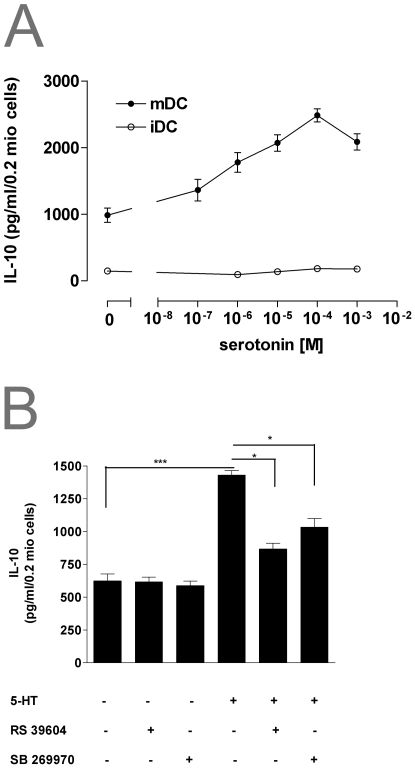
5-HT modulates IL-10 production of human monocyte-derived DCs. (A) Immature and mature DC were stimulated with the indicated concentration of 5-HT concentrations. Supernatants were collected 24 h after stimulation and IL-10 content measured by ELISA. Results are given as mean±SEM (n = 4). (B) Mature DCs were preincubated with 10^−7^ M of the selective5-HTR_4_ antagonist RS39604 or the 5-HTR_7_ antagonist SB269970 30 min prior to stimulation with 5-HT. 24 hours later production of IL-10 was measured by ELISA. Data are means±SE (*n* = 5) *p<0.05, **p<0.01, ***p<0.001.

IL-10 and IL-12p70 are cytokines playing a prominent role in T-cell differentiation [29]. Added together with LPS, 5-HT dose dependently inhibited the production of IL-12p70 (Fig. 4A), whereas IL-10 release was increased (Fig. 5A). This effect was mediated by 5-HTR_4_ and 5-HTR_7_ receptor subtypes, as preincubation of mDC with a combination of the 5-HTR_4_ antagonist RS39604 and the 5-HTR_7_ antagonist SB269970 completely abolished 5-HT induced modulation of IL-10 and IL-12p70 release (Fig. 4/5B). No significant effect of 5-HT on basal IL-10 and IL-12p70 secretion in iDCs could be observed (Fig. 4/5A).

### 5-HT influences the T-cell polarizing capacity of mDCs

**Figure 6:**
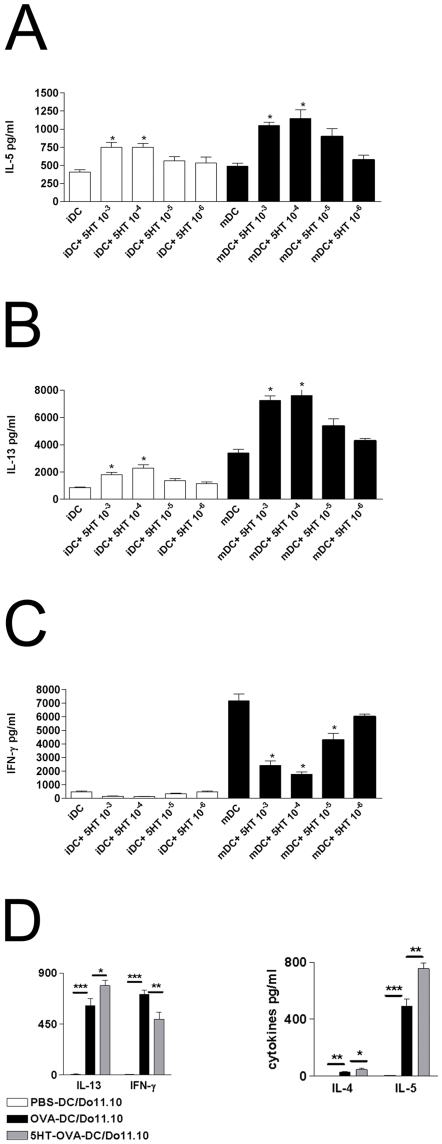
5-HT modulates T-cell priming capacity of human monocyte-derived DCs. Immature DCs were left untreated or stimulated with 5-HT, or were induced to undergo maturation with LPS in the absence or the presence of indicated concentration of 5-HT for 24 h. DCs were then used to prime purified allogeneic CD4+CD45RA+ naive T cells. After 5 days, supernatants from T cells were evaluated for the secretion of IL-5 (A), IL-13 (B) and IFN-γ (C). Results are expressed as mean pg/ml±SD (n = 3). *p<0.05 between cytokines secreted by T cells stimulated with DC treated or not with 5-HT. (D) Naive mice received 10×10^6^ DO11.10 CD4^+^ T-cells intravenously on day-2. On day 0 mice were instilled intratracheally with 10^6^ OVA-pulsed, OVA-pulsed 5-HT treated DCs, or unpulsed DCs. On day4 mediastinal lymph node cells were collected and cultured for 4 days. Four days later, supernatants were harvested and analyzed for the presence of IL-4, IL-5, IL-13, and IFN-γ using commercially available ELISAs .

Starting from our observation that activation of 5-HTR_4_ and 5-HTR_7_ subtypes inhibited the production of IL-12p70, which is an important factor influencing the differentiation of Th1 cells, we next analyzed the quality of primary T cell response induced by DC matured in the presence of different concentration of 5-HT. Naive CD4^+^CD45RA^+^ allogeneic T cells primed with mDC exposed to various concentration of 5-HT during maturation displayed an impaired Th1 and enhanced Th2 polarization, as determined by the increased production of IL-13 and IL-5 as well as down regulation of IFN-γ (Fig. 6). T cells stimulated with 5-HT pre-treated iDC showed an also higher IL-5 and IL-13 secretion (Fig. 6A/B).

### 5-HT induces Th2-priming in vivo

In vivo relevance of Th2-priming induced by 5-HT was investigated in an animal model: female BALB/c mice received a cohort of naive OVA-TCR Tg (DO11.10) T cells intravenously. Two days later, they were injected intratracheally with vehicle treated OVA-pulsed DCs, serotonin-treated OVA-pulsed DCs, or control unpulsed DCs. As shown in figure 6D, mediastinal lymph nodes cells of mice receiving serotonin-pulsed DCs produced higher amounts of IL-4, IL-5, and IL-13 whereas production of IFN-γ was inhibited.

## Discussion

5-HT is one of the major mediators secreted during platelet aggregation or following cross-linking of IgE antibodies on mast cells [2], [3]. Moreover, increasing evidence suggests that 5-HT is involved in the pathogenesis of allergic asthma, as elevated 5-HT levels have been detected in blood and sputum samples of asthmatic patients compared to healthy individuals [19]. Additionally, a negative correlation between serum 5-HT levels and pulmonary function tests was found in asthmatics [19]. Treatment of asthmatic patients with the 5-HT reuptake accelerator tianeptine has been shown to lead to a clinical improvement [30]–[32]. Recently, the functional expression of different 5-HTR subtypes on human monocyte-derived dendritic cells has been reported [25]. In this study, we provide additional evidence that 5-HT is a powerful modulator of dendritic cell function thus being able to enhance allergic airway inflammation.

Thereby, we demonstrated for the first time that 5-HT is a direct chemo-attractant for immature but not mature human DCs. By using the 5-HTR_1B_ antagonist GR 55562 and the 5-HTR_2A_ antagonist ketanserin we could show that both receptor subtypes are involved in the 5-HT-induced migration of iDC. Interestingly, 5-HT has been reported to induce migration of human eosinophils [17] and human aortic smooth muscle cells [33] by selective activation of 5-HTR_2A_-subtypes, while others reported that the receptor subtypes 5HTR_1B_ and 5HTR_1A_ were involved in 5-HT-mediated chemotaxis of human aortic endothelial cells [34] and mast cells [18]. However, during maturation DCs loose the ability to migrate in response to 5-HT. Similar to other well-described mediators of allergic inflammation such as histamine or nucleotides this might be a prerequisite for the departure of DCs to secondary lymphoid organs [35], [36]. As shown by the increased number of DCs in mediastinal lymph nodes following administration of 5-HT, these findings are relevant in vivo. Therefore, 5-HT release during allergen challenge might be important for the recruitment of DCs to the site of inflammation.

Previous studies were able to demonstrate that 5-HT is a potent regulator of cytokine secretion in different kinds of cells. Here we show that 5-HT increase production of the pro-inflammatory cytokine IL-6 in mature DCs via 5-HTR_3_, 5-HTR_4_ and 5-HTR_7_ subtypes. These findings are in accordance with previous studies conducted with human monocytes [14] and airway epithelial cells [13]. Additionally, via activation of 5-HTR_4_ and 5-HTR_7_ receptors, 5-HT up-regulated production of IL-10 by human LPS-matured DCs. Similar effects of 5-HT on IL-10 release have been observed in human LPS-treated monocytes [14], whereas no effect on IL-10 production has been seen in human peripheral blood mononuclear cells treated with 5-HT [37]. IL-12p70 is known as a cytokine favouring Th1-polarisation [38]. According to previous data of our group, IL-12p70 secretion was dose-dependently inhibited by 5-HT [25].

DCs are known to produce different chemokines thereby regulating the traffic of Th1 and Th2 cells into inflamed tissue [39]. Besides its influence on cytokine secretion, stimulation with 5-HT up-regulated production of MDC/CCL22 in both immature and mature DCs, while secretion of CXCL10 was inhibited in mature DCs. 5-HT is known to increase intracellular cAMP levels via activation of 5-HTR_4_ and 5-HTR_7_ receptors [25]. In accordance, cAMP-elevating agents, such as histamine or prostaglandins have similar effects on CCL22 and CXCL10 secretion [40], [41]. CCL22 preferentially attracts Th2 cells, while CXCL10 is a key chemokine for the recruitment of Th1 cells, it can be assumed that DCs exposed to 5-HT (e.g. released during allergen challenge) preferably attract Th2 cells leading to a Th2-dominated microenvironment [42], [43].

Recently Katoh et al reported that 5-HT reduced the capacity of immature DCs to activate allogeneic T-cells [44]. However, the effect of 5-HT on mature DCs has not been investigated in this study. By using unselective 5-HTR_1/6/7_ agonists and antagonists the authors suggest the involvement of 5-HTR_1E_ and 5-HTR_7_ in this cellular response. In contrast, we could show here, that DCs matured in the presence of 5-HT induced a Th2-polarisation in naive CD4^+^CD45RA^+^ T-cells, as demonstrated by downregulation of IFN-y and upregulation of IL-5/IL-13 production consistent with the capacity of 5-HT to increase IL-10 and to inhibit IL-12p70 secretion in maturing DCs. This in vitro observation could be confirmed in an animal model, where 5-HT favoured the outcome of a Th2-response in mediastinal lymph nodes.

DCs are essential for the induction and maintenance of allergic diseases such as bronchial asthma [28], [45]. Here we provide additional evidence that 5-HT can influence human dendritic cell function in a pro-asthmatic way. 5-HT which is released following allergen challenge has chemotactic activity on immature but not mature DCs leading to accumulation of DCs at the site of inflammation. Under the influence of 5-HT DCs secrete larger amounts of IL-6, a cytokine important for airway-remodelling and mucus hypersecretion [46], [47]. By increasing IL-10 and inhibiting IL-12p70 secretion 5-HT influences the T-cell priming capacity of DCs favouring the outcome of a Th2-response [22]. Additionally, up-regulation of CCL22 and down-regulation of CXCL10 release by 5-HT might lead to selective accumulation of Th2-cells into inflamed tissue [43].

In conclusion our data provide further evidence that 5-HT is involved in the pathogenesis of allergic diseases. Therefore, the use of specific 5-HTR antagonists targeting the function of DCs, might be a new therapeutic option in allergy treatment.
